# Energetic Study of Helium Cluster Nucleation and Growth in 14YWT through First Principles

**DOI:** 10.3390/ma9010017

**Published:** 2016-01-02

**Authors:** Yingye Gan, Huijuan Zhao, David T. Hoelzer, Di Yun

**Affiliations:** 1 Department of Mechanical Engineering, Clemson University, Clemson, SC 29631-0921, USA; ygan@clemson.edu; 2 Materials Science & Technology Division, Oak Ridge National Laboratory, PO Box 2008, Oak Ridge, TN 37831-6136, USA; hoelzerd@ornl.gov; 3 School of Nuclear Science and Technology, Xi’an Jiao Tong University, 28 Xi’an Ning West Road, Xi’an 710049, China; diyun1979@xjtu.edu.cn

**Keywords:** helium bubbles, nanostructured ferritic alloys, first principles theory, formation criteria

## Abstract

First principles calculations have been performed to energetically investigate the helium cluster nucleation, formation and growth behavior in the nano-structured ferritic alloy 14YWT. The helium displays strong affinity to the oxygen:vacancy (O:Vac) pair. By investigating various local environments of the vacancy, we find that the energy cost for He cluster growth increases with the appearance of solutes in the reference unit. He atom tends to join the He cluster in the directions away from the solute atoms. Meanwhile, the He cluster tends to expand in the directions away from the solute atoms. A growth criterion is proposed based on the elastic instability strain of the perfect iron lattice in order to determine the maximum number of He atoms at the vacancy site. We find that up to seven He atoms can be trapped at a single vacancy. However, it is reduced to five if the vacancy is pre-occupied by an oxygen atom. Furthermore, the solute atoms within nanoclusters, such as Ti and Y, will greatly limit the growth of the He cluster. A migration energy barrier study is performed to discuss the reduced mobility of the He atom/He cluster in 14YWT.

## 1. Introduction

High performance structural material design has attracted great attention in the fusion reactors industry due to the great demand of the power supply requirement. Researchers have been focused on the development of new structural materials that can maintain excellent material performance under high temperature, high pressure and high irradiation conditions [1]. Especially in such a high energy neutron irradiation environment, large numbers of helium atoms are either directly implanted into or produced internally through (n,α) transmutation reactions in an Fe matrix. Due to the low solubility of He atoms in an iron matrix, these He atoms can be easily trapped with the vacancies from the energetic displacement damage to form He bubbles [2,3,4]. The presence of large He bubbles and their further coalescence are known to promote void swelling, blistering and creep rupture, which drastically decrease the service life and mechanical reliability of the structural materials [5,6,7,8].

Enormous efforts have been made to enhance the helium management and develop high performance structural materials. The high density of dislocations and interfaces in the material are reported to lead to the material resistance enhancement of He-induced damage [9,10,11,12,13,14]. These structures can effectively trap helium, as well as control its diffusion, which mitigate the bubbles to void transformation. A new type of nanostructured ferritic alloy, 14YWT, has been developed with a high density of 2–4 nm sized Y-Ti-O-enriched nanoclusters (NCs) uniformly dispersed within the ultra-fine grain and along grain boundary in the matrix [15,16,17]. 14YWT has exhibited excellent mechanical strength, hardness [17,18] and a low creep rate (six-order lower in magnitude compared to that of the conventional iron alloy) [19,20], at both room temperature and elevated temperature. More importantly, 14YWT has presented great resistance to radiation damage [14,19,21,22,23,24,25,26]. Within the 14YWT matrix, these Y-Ti-O-enriched nanoclusters remain remarkably stable without coarsening, both at elevated temperature (0.92 of the melting temperature) [15,21] and under irradiation condition [17,27,28]. With the amorphous-like atomic configuration, which is coherent with the underlying BCC iron matrix [25,28,29,30], these nanoclusters serve as pins of dislocation migration, therefore leading to the enhancement of the strength and creep resistance. According to recent experiments, the distribution of He bubbles in irradiated 14YWT is extremely homogeneous and has a strong tendency to concentrate at the nanocluster-matrix interface [31]. Through the combined Transmission Electron Microscopy (TEM) and Atom Probe Tomography (APT) data, researchers have indicated that 48.6% of He bubbles are located on the nanoclusters, 14.4% are at the grain boundary, 12.2% are at the dislocations, 4.4% are at the coarse precipitates (Y${}_{2}$Ti${}_{2}$O${}_{7}$ and Ti(N,C)) and the remaining 20% of He bubbles are located within the iron matrix [32]. The size of the bubbles is around 1 nm with the density of 10${}^{23}$ bubbles/m${}^{3}$, similar to the density of NCs. Due to the homogeneous bubble distribution and uniform bubble size, the irradiation-induced damage is significantly reduced in 14YWT. Elucidating the underlying mechanism of such bubble formation and bubble behavior is of great significance to future irradiation-tolerant structural material design.

Researchers have been seeking to understand the formation and growth criteria of helium bubbles within an *α*-Fe matrix under irradiation, both experimentally and computationally. Experimental scientists have observed that vacancies can trap helium atoms and reduce their mobility accordingly [34,35]. From first principles theory calculations, researchers found that He atoms have a tendency to occupy the tetrahedral interstitial positions in an iron matrix [36]. Low migration energy is required for tetrahedral interstitial He atoms to attract each other to form He clusters (bubbles) [4]. He atoms can form strong binding with pre-existing vacancies, and the stability of small He-vacancy clusters has been investigated [37]. Molecular dynamics simulations and multi-scale modeling have also been adopted to investigate the formation and stability of He-vacancy clusters [38,39] and the dislocation mobility of the nodal effect accordingly [40]. To our knowledge, all of the current investigations are focused on the interaction between He and vacancies without a complicated vacancy local environment within an *α*-Fe matrix.

In 14YWT, a high density of pre-existing vacancies is produced during the mechanical alloying process. These pre-existing vacancies present strong binding with oxygen atoms to form the oxygen:vacancy (O:Vac) pairs. The oxygen solubility in an iron matrix can be significantly enhanced [37]. In particular, the composition of Y-Ti-O-enriched naonclusters includes 10% of Y, 40% of Ti and 40% of O [15,21,25]. The nanoclusters can be viewed as an ensemble of interacting O-based units. Each unit consists of an (O:V) pair with Ti and/or Y solute atoms as its nearest neighbor atoms [41]. In order to understand the nucleation, formation and growth of He bubbles within 14YWT, it is critical to understand the interaction of He with the O:Vac-based units, including other solutes (Ti and Y). In this paper, we will present the energetic study of He cluster formation and interaction with vacancy under various local environments in 14YWT through first principles density functional theory calculations. A growth criterion will be proposed to determine the maximum size of the He cluster with one vacancy in 14YWT. In the following, the methodology and computational details will be introduced in Section 2. The results and discussion will be presented in Section 3, including the growth criteria. In Section 4, conclusions will be drawn and further discussions will be presented.

## 2. Methods

In this study, the Vienna *ab initio* Simulation Package (VASP) [42,43,44] (Computational Materials Physics, Sensengasse 8/12, A-1090, Vienna, Austria) is adopted to perform first principles theory calculations. The spin polarized scheme is selected due to the ferromagnetism of *α*-iron. The Projector Augmented Wave (PAW) pseudopotential [45] is chosen to describe the electron-ionic core interaction. The electron exchange and correction is described with Generalized Gradient Approximation (GGA) and Perdew-Burke-Eruzer (PBE) functionals [46]. The Methfessel-Paxton scheme is selected for the smearing function. The cut-off energy is set to 650 eV for all cases. A supercell of 3×3×3 unit cells (54 Fe atoms with the perfect lattice) is adopted for all of the energetic studies in this work. Within the Monkhorst-Pack scheme, we conduct the convergence study with various k-meshes (3×3×3 to 6×6×6) and select a 3×3×3 k-mesh in the following calculations in order to maintain both the energy accuracy of 0.001 eV and the computational efficiency.

**Figure 1:**
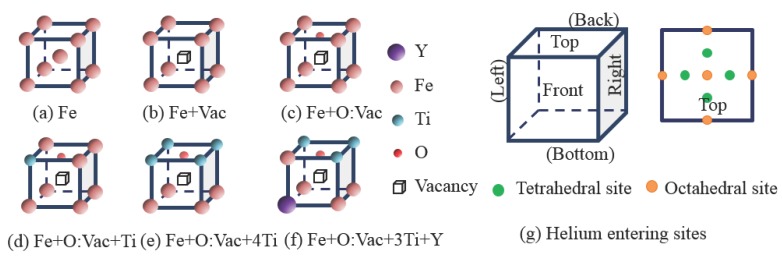
(**a**)–(**f**) Schematic atomic configuration of the reference units; (**g**) The possible entering sites of He on the reference unit during the He cluster growth. The six planes of the reference unit are named top, front, right, bottom, back and left. On each plane, the 5 octahedral sites and 4 tetrahedral sites are presented.

Since most of the He bubbles are located on the nanoclusters, at the grain boundary and within the 14YWT iron matrix [32], we select six reference units to investigate the He cluster nucleation, formation and interaction within the 14YWT matrix, shown in Figure 1. Figure 1a,b denotes the reference units with a perfect lattice and a single vacancy, respectively. Figure 1c is the reference unit with the O:Vac pair. Figure 1d is the reference unit of an O:Vac pair with one Ti atom, which is the most stable state of the O:Vac pair in the 14YWT matrix [41]. Figure 1e,f is the two reference units, which represent the two major local environments of the O:Vac pair within the nanoclusters [41]. We do not consider the charge distribution near the vacancy in this study, since the entire supercell is set to be neutral [33]. In order to energetically investigate the He cluster nucleation and formation behavior, we replace the center unit cell in the super-cell with the reference unit cell listed in Figure 1, respectively, and add He atoms one by one into the reference unit through all 42 tetrahedral and octahedral interstitial sites, as shown in Figure 1g. For each configuration, the symmetry of these entering sites has been considered in order to limit the number of calculations. Since the He atom can move easily between interstitial sites within an iron matrix due to the low migration energy barrier (0.06 eV) [4], the newly-added He atom can easily migrate within the super-cell in order to achieve the equilibrium energy state. Due to the various entering sites, multiple numbers of equilibrium configurations can be achieved. In the following discussion, the helium sample entering site (SES) is described with the format of αβγ. *α* is the helium entering plane, which can be F (front), K (back), T (top), B (bottom), L (left), R (right) and A (all). *β* is the helium interstitial site, which can be T (tetrahedral), O (octahedral) and A (all). *γ* is the helium interstitial position on each plane, which can be A (all) and C (center), and the plane close to He atom: T (top), B (bottom), L (left), R (right), F (front) and K (back).

In this study, all of the calculations are based on the constant volume condition with the lattice constant $a_{0}=2.83\;$Å for a perfect BCC iron lattice. To understand the He cluster formation and growth, we focus on the formation energy and the He trapping energy of each individual configuration. The formation energy is defined as:

(1) $$E_{f}\left(\mathrm{ref}+n\mathrm{He}\right)=E\left(\mathrm{ref}+n\mathrm{He}\right)-E\left(\mathrm{ref}\right)-nE\left(\mathrm{He}\right)$$

where ref+nHe denotes the reference unit with *n* number of He atoms in the super-cell; subscript f denotes the formation energy. E(He) = -0.0046 eV is the energy of an isolated He atom in the vacuum space [47]. During the He cluster formation, the He trapping energy is an important parameter to determine whether the *n*-th He atom can be trapped into the He${}_{\left(n-1\right)}$ cluster. For the accumulation of the *n*-th He atom, the He trapping energy is defined as:

(2) $$E_{\mathrm{trap}}\left(\mathrm{ref}+n\mathrm{He}\right)=E_{f}\left(\mathrm{ref}+n\mathrm{He}\right)-E_{f}\left(\mathrm{ref}+\left(n-1\right)\mathrm{He}\right)-E_{f}\left({\mathrm{He}}_{\mathrm{tetra}}\right)$$

where $E_{f}\left({\mathrm{He}}_{\mathrm{tetra}}\right)$ is the formation energy of a tetrahedral interstitial He within the perfect Fe lattice.

## 3. Results and Discussion

### 3.1. He Interaction with a Vacancy and the O:Vac Pair

**Figure 2:**
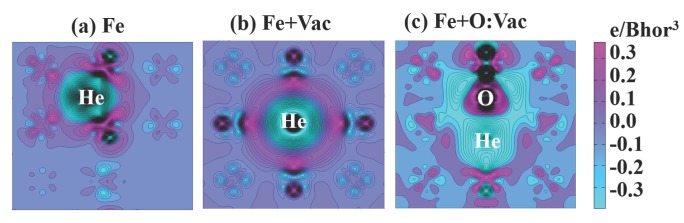
The change in the charge density of Fe on the (0 2 0) plane due to the He atom: (**a**) A tetrahedral interstitial He in a perfect Fe lattice; (**b**) a He trapped at the vacancy site of a Fe lattice; and (**c**) a He trapped with the O:Vac (Vac, vacancy) pair in a Fe lattice.

In our calculation, the formation energy is $E_{f}\left(\mathrm{Vac}\right)$ = 2.15 eV for the vacancy, $E_{f}\left({\mathrm{He}}_{\mathrm{tetra}}\right)=4.63$ eV for the tetrahedral interstitial He and $E_{f}\left({\mathrm{He}}_{\mathrm{octa}}\right)$ = 4.84 eV for the octahedral interstitial He. The formation energy of He at the substitutional site is $E_{f}\left({\mathrm{He}}_{\mathrm{sub}}\right)$ = 4.41 eV. Without a vacancy, the He atom prefers the tetrahedral interstitial site with a lower formation energy; otherwise, the He atom is trapped in the vacancy as a substitution. These results match well with recently-published data [47], which are also based on VASP calculations. These results are slightly higher than other published data [4,36,48,49], mainly due to different Density Functional Theory (DFT) solvers and setups. However, these calculations reach the same conclusion qualitatively. The formation energy of He to occupy the pre-existing vacancy site is only $E_{f}^{\mathrm{Vac}}\left(\mathrm{He}\right)$ = 2.26 eV, half of that for the tetrahedral interstitial site. Thus, with the pre-existing vacancy, the He atom can be easily trapped at the vacancy site. Since most of the pre-existing vacancies are occupied by oxygen atoms in 14YWT, we calculate the interaction between the He atom and the O:Vac pair. The formation energy of He in this configuration is calculated to be $E_{f}^{O:\mathrm{Vac}}\left(\mathrm{He}\right)$ = 2.42 eV, similar to $E_{f}^{\mathrm{Vac}}\left(\mathrm{He}\right)$. For the reference units shown in Figure 1a–c, the charge density difference of Fe due to the addition of He atoms is shown in Figure 2, respectively. For interstitial He in Figure 2a, the He atom and its first neighboring Fe atom are strongly polarized as a result of the interaction. For substitutional He shown in Figure 2b, no polarization can be observed [49]. In Figure 2c, significant polarization represents the strong interaction between He and the O:Vac pair. Therefore, the effect of pre-occupied oxygen at the pre-existing vacancy site on the He trapping behavior exists, but is very limited. The He atom still displays strong affinity to the O:Vac pair in 14YWT.

### 3.2. Energetics of He Clusters with a Vacancy and the O:Vac Pair

**Figure 3:**
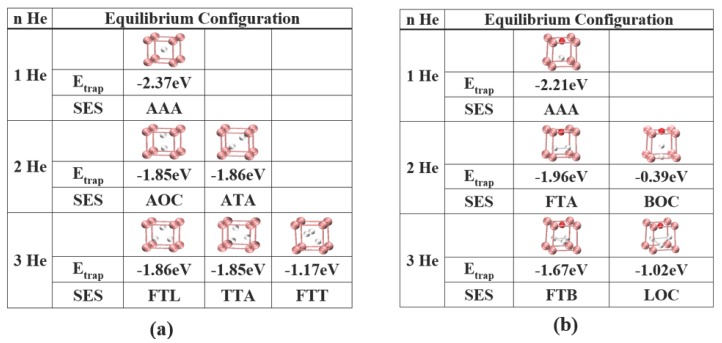
The equilibrium configurations and corresponding He trapping energy of the He cluster with a reference unit of: (**a**) Fe + Vac; and (**b**) Fe + O:Vac, respectively. The Fe atom and the He atom are presented as pink and white, respectively.

Let us denote He cluster as He${}_{n}$X, where *n* is the number of He atoms in the cluster and X represents the reference unit listed in Figure 1. Since the pre-existing vacancies are occupied by oxygen atoms to form a strong bonded O:Vac pair in 14YWT [37], we first investigate the He cluster formation with reference units of X = Fe + Vac and X = Fe + O:Vac, shown in Figure 3a,b, respectively. He atoms are gradually added into the reference unit from the interstitial sites shown in Figure 1g. Depending on the entering sites, various equilibrium configurations will be reached. For simplification, we only list some of the favorable sample entering sites. We also present the corresponding $E_{\mathrm{trap}}$. Since multiple equilibrium configurations of He${}_{n}$X clusters are reached with n≥2, we adopted the lowest $E_{f}$ in He${}_{\left(n-1\right)}$X clusters for $E_{\mathrm{trap}}$ calculations by using Equation (2). The non-equilibrium configurations and equilibrium configurations with positive $E_{f}$ are not listed here.

It is clear to observe that with only a vacancy in the reference unit (X = Fe + Vac), the first He atom can be trapped in the vacancy site with strong He trapping energy $E_{\mathrm{trap}}$ = −2.37 eV. More He atoms can be easily attracted to the vacancy site with stable He trapping energy. For n≥2, various equilibrium configurations are achieved. Some of the symmetric configurations are identical to the published results [38]. However, the difference between He trapping energy is limited. There is an exception with n=3, where the third configuration can be achieved with $E_{\mathrm{trap}}$ = −1.17 eV, 37% higher than that of the other equilibrium configurations. However, the possibility of the He${}_{3}$X cluster to reach the third configuration is limited (4 out of 84). With the O:Vac pair in the reference unit (X = Fe + O:Vac), the first He atom can be trapped with the O:Vac pair with $E_{\mathrm{trap}}$ reduced by only 6%, compared to that in the X = Fe + Vac case. When n≥2, multiple equilibrium configurations can be reached. The $E_{\mathrm{trap}}$ presents significant fluctuation. With the existence of an oxygen atom, the $E_{\mathrm{trap}}$ for the 2 He atom case is even lower than that in the X = Fe + Vac case. With the increasing of *n*, the $E_{\mathrm{trap}}$ is significantly reduced.

**Figure 4:**
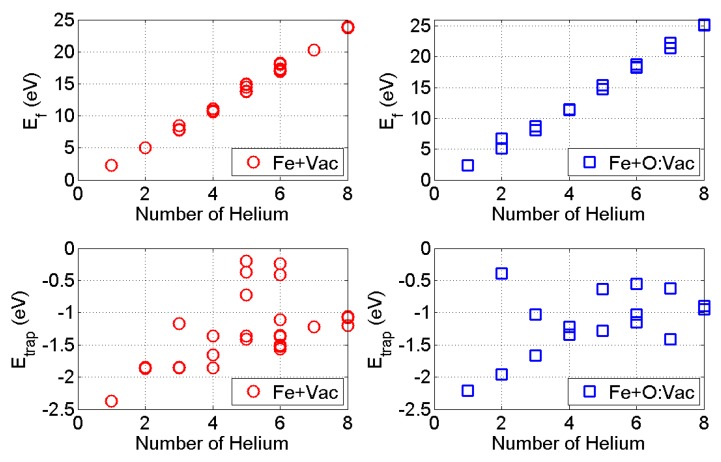
Formation energy and He trapping energy variation with *n* in the He${}_{n}$X cluster: X = Fe + Vac (red circles); and X = Fe + O:Vac (blue squares).

We have calculated the growth of He${}_{n}$X clusters with *n* up to 8. The formation energy and He trapping energy variations with the number of He atoms in the He${}_{n}$X clusters are presented in Figure 4. In both cases, the formation energy of the He${}_{n}$X cluster increases monotonically with the number of He atoms, consistent with previous Molecular Dynamics (MD) calculations [38] and DFT calculations [47]. The slope of $E_{f}$ represents the energy penalty rate for the growth of the small He${}_{n}$X cluster. For the Fe + Vac case, the formation energy increases with a slope of 3.08±0.02 eV/atom. For the Fe + O:Vac case, the formation energy increases with a slope of 3.22±0.01 eV/atom. In particular, both cases have a lower energy penalty rate (≤3.00 eV/atom) with n≤4 and a higher energy penalty rate (≥3.40 eV/atom) when n≥6. This reflects the higher energy requirement for larger He${}_{n}$X clusters to form. In both cases, the He trapping energies present an ascending trend with the number of He atoms, while remaining negative. With the criterion of negative He trapping energy, the He${}_{n}$X clusters can continue growth when *n* = 8.

### 3.3. Energetics of He Clusters in 14YWT

**Figure 5:**
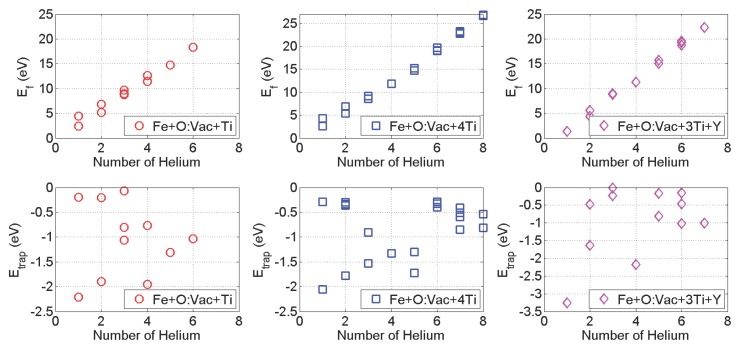
Formation energy and He trapping energy variation with *n* in He${}_{n}$X clusters: X = Fe + O:Vac + Ti (red circles), X = Fe + O:Vac + 4Ti (blue squares), and X = Fe + O:Vac + 3Ti + Y (magenta diamonds).

In 14YWT, the major local environments of the O:Vac pair within the matrix and the nanoclusters are represented in Figure 1d–f, respectively [41]. It is important to understand the He${}_{n}$X clusters’ formation and growth mechanism with other solute atoms, such as Ti and Y, located within the O:Vac local environment. We adopt the same methodology discussed in the previous section to investigate the growth of He${}_{n}$X clusters, with X representing Fe + O:Vac + Ti, Fe + O:Vac + 4Ti and Fe + O:Vac + 3Ti + Y, respectively. The formation energy and He trapping energy are listed in Figure 5. In all three cases, the formation energy of the He${}_{n}$X cluster remains the monotonically increasing trend with the number of He atoms.

However, the slope of $E_{f}$ is 3.33±0.11 eV/atom and 3.49±0.01 eV/atom in the Fe + O:Vac + 4Ti and Fe + O:Vac + 3Ti + Y cases, respectively. They are much higher than that of the Fe + Vac and Fe + O:Vac cases. Since the slope of $E_{f}$ represents the energy penalty rate for the growth of the He${}_{n}$X cluster, this means more energy is required for the He${}_{n}$X clusters’ growth near the nanocluster interface than that within the matrix. The He trapping energy variation with *n* presents a different behavior compared to that of the Fe + Vac and Fe + O:Vac cases. With other solutes in the local environment, the magnitude of the lowest He trapping energy is greatly reduced, compared to those in the Fe + Vac and Fe + O:Vac cases.

**Figure 6:**
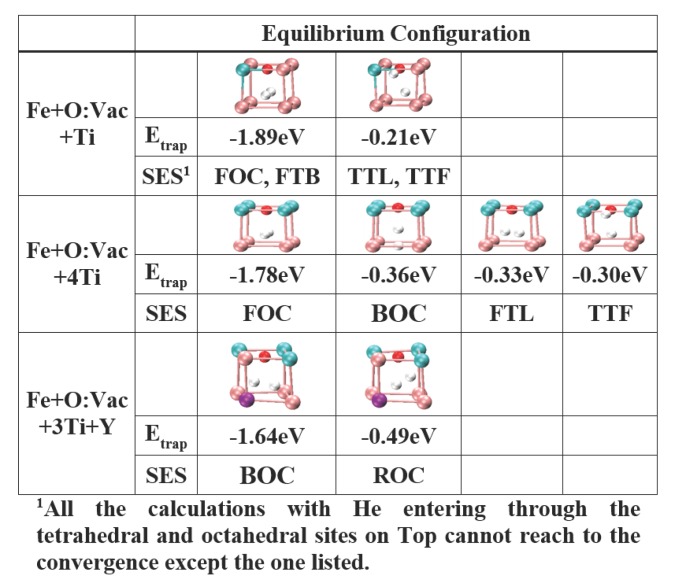
The equilibrium configurations and corresponding He trapping energy of He${}_{2}$X clusters with X = Fe + O:Vac + Ti, X = Fe + O:Vac + 4Ti and X = Fe + O:Vac + 3Ti + Y.

In particular, the divergence of He trapping energy in the small *n* cases brings the attention of the effect of He entering sites to the growth of the He${}_{n}$X cluster. Let us take *n* = 2 as an example. Figure 6 lists the equilibrium configurations of He${}_{2}$X with X = Fe + O:Vac + Ti, Fe + O:Vac + 4Ti and Fe + O:Vac + 3Ti + Y, respectively. It can be clearly identified that He prefers to enter the He${}_{n}$X clusters from the sites away from the solute atoms (Ti and Y). Adopting the X = Fe + O:Vac + 4Ti case as an example, the He entering sites that are close to the Ti atoms (the interstitial sites on the top plane) can only result in interstitial He atoms in the neighboring unit cells (the fourth configuration). For all of the other interstitial sites, the second He atom can be easily attracted by the reference unit and equilibrium at the most energy favorable site shown in the first column. In other words, even though the O:Vac pair performs as the He sink, the Ti and Y atoms impose a blocking effect during the growth of He clusters, preventing the close-by He atom from joining the He cluster centered at the O:Vac pair. More importantly, it is difficult for He atoms to enter the nanocluster and nucleate the He cluster inside the nanocluster, which contains 40% Ti and 10% Y atoms[15,21,25]. Therefore, this explains the experimental observations that most of the He bubbles are formed next to the nanoclusters without penetrating inside [32].

### 3.4. Formation and Growth Criteria of the He Cluster in 14YWT

Researchers have proposed various He bubble growth criteria in an *α*-Fe matrix. Previous studies have reported a maximum He${}_{4}$ cluster with DFT simulation [50] and a maximum He${}_{6}$ [51] cluster with MD simulation in a vacancy-free iron matrix. Recently, Dr. Lu’s group proposed that up to eight He atoms can be trapped at a single vacancy in an *α*-iron matrix. In order to capture more He atoms, the vacancy has to emit Frenkel pairs to release the substantial stress building on the surrounding Fe lattice [47]. During the He accumulation within the local environment of the vacancy/O:Vac pair, significant pressure has been produced, causing significant distortion of the local lattice structure. After the local lattice structure reaches its elastic instability, it must conduct plastic deformations (phase transition, dislocation nucleation, *etc*.) in order to release the pressure, therefore providing more space for the continued growth of the He cluster. Herein, we propose to adopt the elastic instability strain of a perfect BCC Fe lattice as the criteria for the He cluster growth at the vacancy site.

In order to determine the elastic instability strain of the perfect BCC Fe lattice, the tri-axial strain-stress tensile test is performed along the 〈001〉 direction. The ideal strength is reached at 15% strain, matching well with the published data [52]. Continuing to load after reaching 15% strain will associate the BCC iron lattice with an elastic instability along the Bain path from BCC to FCC [52]. Therefore, we adopted 15% as the maximum bond strain to evaluate the maximum number of He atoms in the He cluster growth at the reference units in Figure 1.

**Figure 7:**
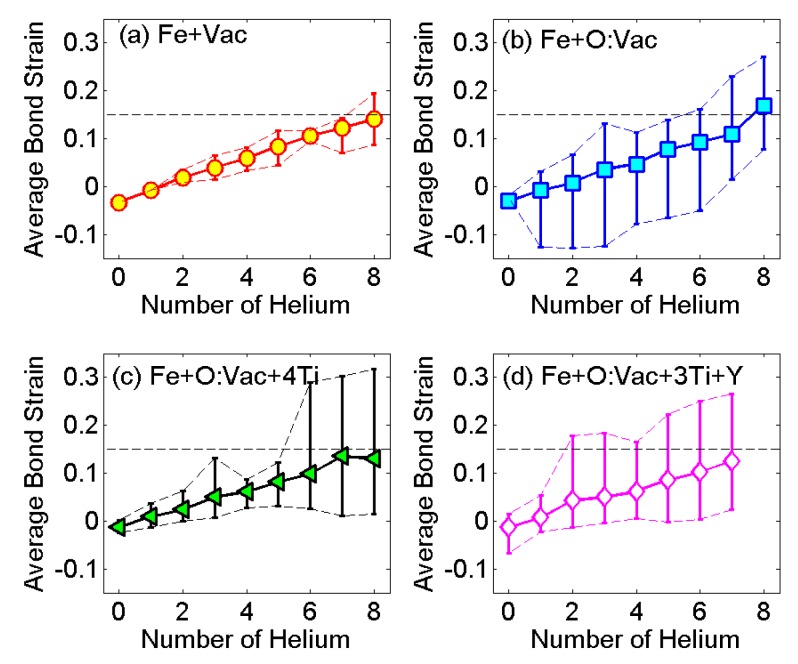
Bond length variations in various reference units: (**a**) Fe+Vac; (**b**) Fe+O:Vac; (**c**) Fe+O:Vac+4Ti; and (**d**) Fe+O:Vac+3Ti+Y.

To investigate the local distortion and maximum size of the He cluster, the bond strain variations with the number of He in He${}_{n}$X clusters are presented in Figure 7. The solid lines with a symbol represent the average bond strain within the reference unit of the super-cell (12 bonds in each reference unit). The dashed lines represent the maximum and minimum bond strain at the corresponding condition. It is clear that with a single vacancy in the iron matrix, the local bond distortion is limited. The maximum number of He atoms in the He cluster is *n* = 7, since the maximum bond strain exceeds 15% at *n* = 8. This result is closely consistent with the recent publication in which the growth criteria of a He cluster with a single vacancy is determined by the emission of Frenkel pairs [47]. With the O:Vac pair, local bond distortion becomes significant. The maximum number of He atoms in the He cluster is *n* = 5, shown in Figure 7b. With the major local environment of the O:Vac pair within the nanoclusters (Figure 1d,f), the maximum number of He atoms is reduced to *n* = 5 and *n* = 1 for Fe + O:Vac + 4Ti case and Fe + O:Vac + 3Ti + Y case, respectively. The bond distortion increases dramatically after the limit is reached. The maximum bond strain variation can exceed 25%. It is obvious that the existence of other solutes can reduce the size of He cluster associated with a single vacancy, especially the Y atom.

**Figure 8:**
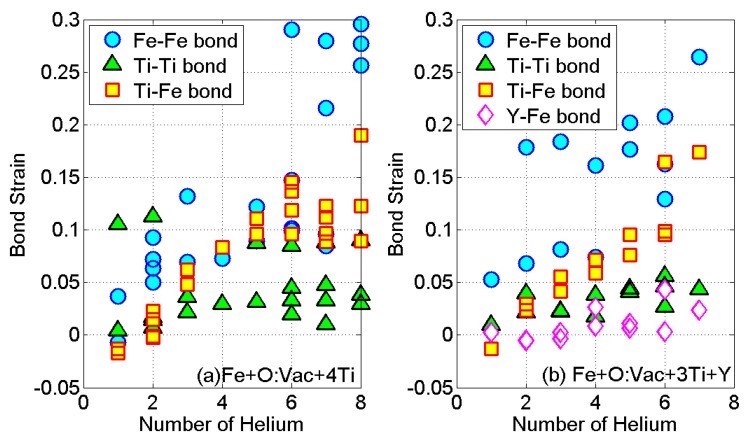
(**a**) bond strain variation of the reference unit of Fe + O:Vac + 4T; (**b**) bond strain variation of the reference unit of Fe + O:Vac + 3Ti + Y.

In order to understand the significant local distortion in Figure 7c after *n* = 6 and Figure 7d after *n* = 2, we further plot the Fe-Fe, Ti-Ti, Ti-Fe and Y-Fe bond strain variations with the number of He atoms, shown in Figure 8. Fe-Fe bonds are much more severely distorted by the He cluster than the other bonds. The strain on Ti-Ti bonds and Y-Fe bonds remains stable during the growth of He cluster. Such results implicate the preference in the nucleation site of the He${}_{n}$X cluster from the aspect of bond strain variation. He atoms tend to avoid the stronger Ti-Ti bonds during the nucleation process. A He bubble can be easily nucleated on nanoclusters due to the high density of the O:Vac pair, but will grow in the near-by matrix at the NC-matrix interface without penetrating the NCs. This theoretical finding is consistent with the experimental observation in reference [32].

As we discussed, the initial nucleation and growth of the He bubble mainly depends on the interaction between the He cluster and a single vacancy under various local environments. However, the size of He bubbles after irradiation is also related to the mobility of He atoms in the He${}_{n}$X cluster. Interstitial He atoms have a very low migration energy of 0.06 eV. The migration of substitutional He is found to be governed by the migration of the Vac-He-Vac complex, with an energy barrier of 1.1 eV [4]. In order to understand the effect of other solutes on the He/He${}_{n}$X cluster migration in 14YWT, we adopted the nudged elastic band method (NEB) [53] to compute the migration energy of He between the Reference Unit (b) and the Reference Units (b, c, e) shown in Figure 1. We found that the migration energy barrier of the He atom in the Fe + Vac → Fe + O:Vac + 4Ti path is 0.5 eV higher than that in the Fe + Vac → Fe + Vac path. The migration energy barrier of He atom in the Fe + Vac → Fe + O:Vac path is 0.2 eV higher than that in the Fe + Vac → Fe + Vac path. Although more quantitative investigations should be done to fully understand the migration behavior of He atoms/He${}_{n}$X clusters in 14YWT, qualitatively, we find it is more difficult for He atoms to diffuse between two trapping sites with the presence of other solutes. The coalescence of the small He clusters into into multiple vacancy-induced He bubbles might be hindered because of the barriers provided by these solutes in the He atom diffusion process. Such impedance produced by these solutes can be a crucial factor in the He bubble size-controlling mechanism in 14YWT.

## 4. Conclusions

In summary, first principles calculations have been conducted to understand the He cluster nucleation, formation and growth behavior within 14YWT. Six types of reference units are considered, representing the major local environment within the matrix and nanocluster of 14YWT. The O:Vac pair presents similar He trapping behavior with a single vacancy. The He entering site plays an important role in the formation of the He cluster with the presence of other solutes, such as Ti and Y. He atoms tend to enter the He cluster through the directions away from Ti and Y atoms. At the same time, He clusters show the tendency to expand in the directions away from Ti and Y atoms. By investigating various local environments of the vacancy, we find that the energy cost of He cluster formation is increasing with the presence of other solutes in the reference unit. In order to determine the maximum number of He atoms near one vacancy site, a growth criterion is proposed based on the elastic instability strain of the perfect iron lattice. We find that up to seven He atoms can be trapped at a single vacancy, consistent with a recent finding based on the emission of Fe Frenkel pairs [47]. With the proposed criteria, we find that the maximum number of He atom in the He cluster is reduced to five if the vacancy is pre-occupied by an oxygen atom. Furthermore, the solutes within nanoclusters, such as Ti and Y, will greatly limit the growth of the He cluster. From the migration energy barrier calculation, we think the ultra-fine He bubble size in 14YWT can greatly depend on the reduced mobility of He atom/He clusters caused by the solutes (O, Ti and Y) in the local environment. Our theoretical understanding of the He cluster formation and growth is consistent with the recent experimental observations [32].
